# Differences in lobar microbleed topography in cerebral amyloid angiopathy and hypertensive arteriopathy

**DOI:** 10.1038/s41598-024-54243-1

**Published:** 2024-02-15

**Authors:** Pin-Yan Kuo, Hsin-Hsi Tsai, Bo-Ching Lee, Pu-Tien Chiang, Chia-Ju Liu, Ya-Fang Chen, Jiann-Shing Jeng, Ruoh-Fang Yen, Li-Kai Tsai

**Affiliations:** 1 Department of Medical Education, National Taiwan University Hospital, Taipei, Taiwan; 2 Department of Neurology, National Taiwan University Hospital, Taipei, Taiwan; 3 Department of Medical Imaging, National Taiwan University Hospital, Taipei, Taiwan; 4 Department of Neurology, National Taiwan University Hospital Bei-Hu Branch, Taipei, Taiwan; 5 Department of Nuclear Medicine, National Taiwan University Hospital, Taipei, Taiwan; 6 Department of Neurology, National Taiwan University Hospital Hsin-Chu Branch, Hsin-Chu, Taiwan

**Keywords:** Cerebral microbleed, Cerebral amyloid angiopathy, Hypertension, Pittsburgh Compound B, Intracerebral hemorrhage, Small vessel disease, Cerebrovascular disorders, Stroke

## Abstract

Lobar cerebral microbleeds are a characteristic neuroimaging finding in cerebral amyloid angiopathy (CAA) but can also be found in hypertensive arteriolosclerosis. We aimed to investigate whether CAA is more associated with intracortical lobar microbleeds than hypertensive arteriosclerosis. Ninety-one survivors of spontaneous intracerebral hemorrhage with at least one lobar microbleed were included and underwent brain MRI and amyloid PET. We categorized lobar microbleeds as intracortical, juxtacortical, or subcortical. We assessed the associations between the lobar microbleed categories and microangiopathy subtypes or cerebral amyloid load based on the Pittsburgh Compound-B PET standardized uptake value ratio (SUVR). Patients with CAA had a higher prevalence of intracortical lobar microbleeds (80.0% vs. 50.8%, *P* = 0.011) and lower prevalence of subcortical lobar microbleeds (13.3% vs. 60.1%, *P* < 0.001) than patients with hypertensive arteriolosclerosis. Strictly intracortical/juxtacortical lobar microbleeds were associated with CAA (OR 18.9 [1.9–191.4], *P* = 0.013), while the presence of subcortical lobar microbleeds was associated with hypertensive arteriolosclerosis (OR 10.9 [1.8–68.1], *P* = 0.010). Amyloid retention was higher in patients with strictly intracortical/juxtacortical CMBs than those without (SUVR = 1.15 [1.05–1.52] vs. 1.08 [1.02–1.19], *P* = 0.039). Amyloid retention positively correlated with the number of intracortical lobar microbleeds (*P* < 0.001) and negatively correlated with the number of subcortical lobar microbleeds (*P* = 0.018). CAA and cortical amyloid deposition are more strongly associated with strictly intracortical/juxtacortical microbleeds than subcortical lobar microbleeds. Categorization of lobar microbleeds based on anatomical location may help differentiate the underlying microangiopathy and potentially improve the accuracy of current neuroimaging criteria for cerebral small vessel disease.

## Introduction

Cerebral small vessel disease (SVD) refers to a group of vascular pathologies with various causes that affect the small vessels of the brain and lead to both ischemic and hemorrhagic consequences^1^. The two major forms of SVD, cerebral amyloid angiopathy (CAA) and hypertensive deep perforator arteriopathy, are the main etiologies of spontaneous intracerebral hemorrhage (ICH) and cerebral microbleeds (CMBs). CAA occurs due to progressive deposition of β-amyloid in the walls of small-to-medium-sized vessels in the cerebral cortex and overlying leptomeninges, while hypertensive arteriolosclerosis (HA) predominantly involves arteriosclerotic pathologies in the deep-seated vessels that stem directly from the large vessels as arterial perforators^2^.

Clinically, the differentiation of CAA and HA is mainly based on supratentorial neuroimaging markers, especially the distinct hemorrhagic distribution patterns. According to the pathologically validated Boston criteria, patients who present with lobar ICH with strictly lobar CMBs and/or cortical superficial siderosis are strongly considered to have CAA^3–5^. On the contrary, HA predominantly manifests as acute and chronic ICH and/or CMBs in deep brain regions^6^; however, the arteriopathy can also extend to the lobar areas and result in hemorrhagic lesions in both the lobar and deep regions^7–9^. Both clinical and pathological evidence indicate CAA and HA underlie the pathogenesis of lobar CMBs; however, there is a scarcity of published data regarding the topographical differences in lobar CMBs between these two main forms of SVD. Furthermore, the diagnostic value of lobar CMBs in individuals without ICH is limited^10^. Thus, a more refined approach to categorize lobar CMBs is needed to improve clinical diagnosis of the underlying microangiopathy in SVD.

Based on the knowledge that CAA more frequently affects the superficial vessels of the brain while HA mainly involves the deeper perforators, we tested our hypothesis that cerebrovascular amyloid pathology is more strongly related to intracortical lobar CMBs whereas HA is more strongly related to subcortical lobar CMBs.

## Methods

### Patient selection

**Figure 1:**
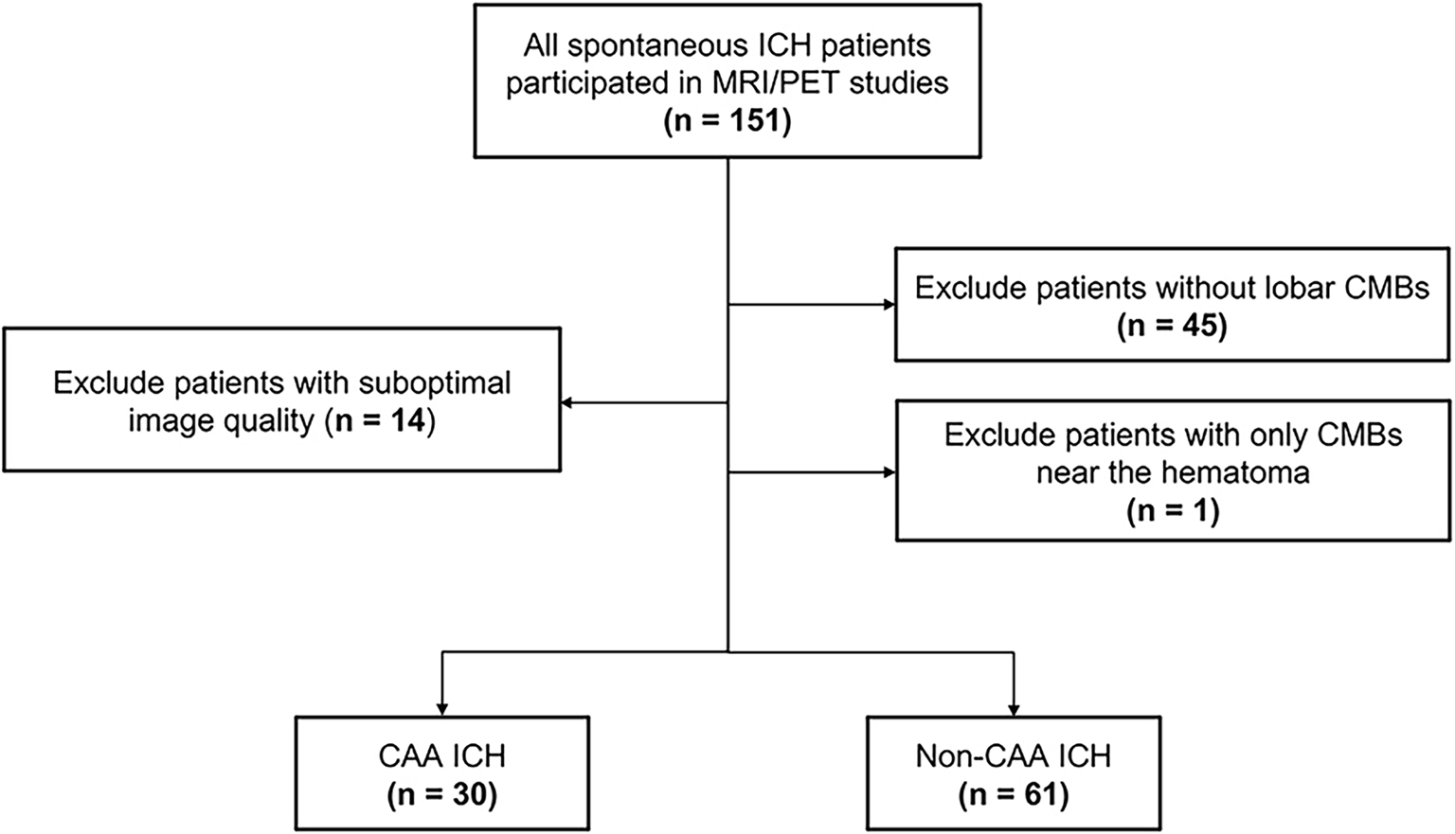
Flowchart of patient enrollment. Of the 151 survivors of spontaneous intracerebral hemorrhage (ICH) who agreed to participate in the study, we excluded patients without any lobar cerebral microbleeds (CMBs; *n* = 45), patients for whom image quality was suboptimal (*n* = 14), and patients with CMBs only close to the hematoma (*n* = 1). In total, 30 patients with cerebral amyloid angiopathy (CAA)-related ICH (defined as strictly lobar ICH and/or CMBs) and 61 patients with non-CAA ICH (mixed deep and lobar ICH/CMBs) were included in the analysis.

We prospectively recruited patients who had suffered symptomatic spontaneous ICH for brain MRI and ^11^C-Pittsburgh compound B (PiB) PET scans at NTUH between September 2014 and October 2022 (Fig. 1)^11,12^. We excluded patients with potential causes of secondary hemorrhage, including trauma, structural or vascular lesions, brain tumors, severe coagulopathy due to systemic disease or medication, or patients who suffered ischemic stroke with hemorrhagic transformation. Patients were excluded if they could not tolerate imaging studies, including patients with a poor ability to cooperate, hemodynamic instability, or an implanted cardiac pacemaker. A total of 151 patients with ICH fulfilling the enrollment criteria agreed to participate in this study and received brain MRI and PiB PET scans (Fig. 1). We excluded patients who did not have any lobar CMBs (*n* = 45) or only had lobar CMBs adjacent to the previous hematoma (*n* = 1). Patients for whom the imaging quality was suboptimal were also excluded (*n* = 14). Thus, a final sample of 91 patients were included in this analysis (Fig. 1). As previously described, patients with lobar ICH(s) involving the cerebral cortex and underlying white matter with strictly lobar CMBs and/or cSS were defined as having CAA (*n* = 30) according to the Boston criteria 1.5^4,5^, while patients were defined as having HA (*n* = 61) if the ICH and CMBs were located in both the lobar and deep regions, as we previously proposed^11,12^. Baseline clinical data collection was performed by the investigators through a comprehensive review of medical records and interviewing each participant. The following clinical variables were systematically recorded for each patient: age, sex, presence of chronic hypertension (defined as clinical diagnosis of hypertension with more than 3 months of prescription of anti-hypertensive agents), classes of anti-hypertensive medication, diabetes mellitus, hypercholesterolemia, history of ICH and ischemic stroke, and creatinine clearance value (represented by estimated glomerular filtration rate).

### MRI acquisition and analysis

Brain MRIs were obtained using a 3-Tesla scanner (Siemens Verio, TIM, or mMR, Siemens Medical Solutions, Malvern, PA, USA). The imaging protocols included T1-weighted imaging, T2-weighted imaging, fluid-attenuated inversion recovery imaging, susceptibility weighted imaging (SWI), diffusion-weighted imaging, apparent diffusion coefficient mapping, and 3D T1-weighted MPRAGE (Magnetization Prepared Rapid Acquisition Gradient Echo) imaging in 1-mm-slice thicknesses. CMBs were defined as lesions with homogeneous round signal loss and a diameter less than 10 mm on SWI and were categorized as lobar or deep based on well-validated criteria^13,14^.

**Figure 2:**
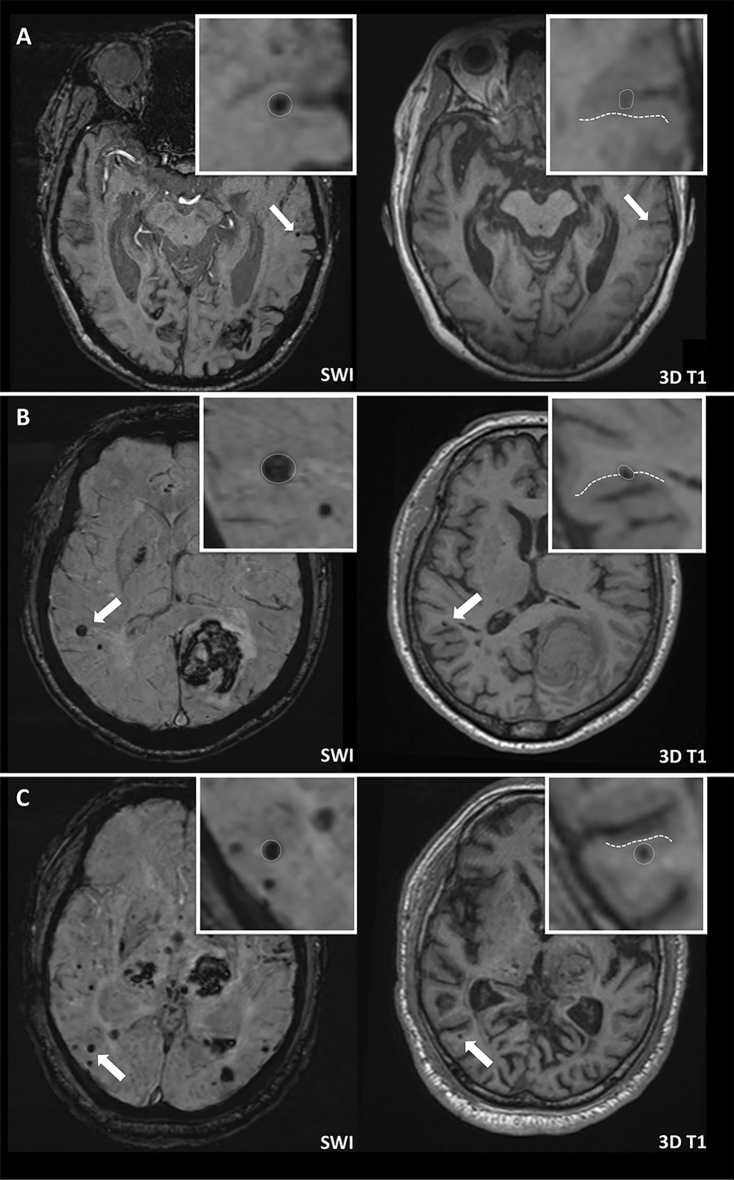
Categorization of lobar CMBs. The location of each lobar CMB was categorized using SWI and the corresponding 3D T1-weighted images. (**A**) Intracortical CMB: the lobar CMB is completely located in the gray matter of the cortex. (**B**) Juxtacortical CMB: the CMB is located on the border of a gray-white matter junction. (**C**) Subcortical CMB: the CMB is completely located in white matter. The borders of the cortex are outlined with dotted lines.

Each lobar CMB was first identified by reviewing SWI sequences. However, due to the limitations of SWI with respect to anatomical discrimination, lobar CMBs were further categorized as intracortical, juxtacortical, or subcortical based on their relative location to the cortex using the corresponding T1-weighted multiplanar reconstruction images, as shown in Fig. 2. Intracortical CMBs were defined as CMBs completely located in the gray matter of the cortex (Fig. 2A); juxtacortical CMBs were defined as CMBs located on the border of gray-white junctions (Fig. 2B); subcortical CMBs were defined as lobar CMBs located in the white matter without reaching the cortex (Fig. 2C). Strictly intracortical/juxtacortical lobar CMBs were defined as having no lobar CMB in the subcortical white matter. All MRI scans were independently rated by two investigators (P.-Y. K., 3-year reading experience and P.-T. C., 6-year reading experience) to determine the inter-rater reliability for the presence/absence of intracortical and subcortical CMBs. If there was disagreement between the two readers, the same investigators reached a consensus decision after discussion.

Other MRI markers related to cerebral SVD were evaluated based on the Standards for Reporting Vascular Changes on Neuroimaging criteria^8,14,15^. Briefly, the presence and number of CMBs and cortical superficial siderosis were evaluated on axial SWI sequences, as previously described^13,16^. The number of CMBs was calculated in the lobar (i.e., the frontal, temporal, parietal, occipital, and insular cortices) and deep regions (i.e., the brainstem, BG, thalamus, internal capsule, external capsule, corpus callosum, and deep periventricular white matter). Cerebellar CMBs were not evaluated in the current study. Lacunes were evaluated in the supratentorial region and defined as ovoid or round, subcortical, fluid-filled cavities ranging in diameter from 3 to 15 mm^17,18^. WMH volume was calculated based on fluid-attenuated inversion recovery imaging using a semi-automated measure, as we previously described^8,19^. The volume estimates were performed in the ICH-free hemisphere and multiplied by two. MRI-visible perivascular spaces (PVS) were evaluated on T2-weighted imaging and defined as sharply delineated structures measuring < 3 mm following the course of perforating or medullary vessels^20^. The number of PVS were counted in the centrum semiovale (CSO) and basal ganglia (BG) on the side of the brain with more severe involvement. The severity of PVS was rated using a validated visual scale (0 = none, 1 =  < 10, 2 = 11–20, 3 = 21–40, and 4 =  > 40)^20,21^. According to a previously proposed method, we pre-specified a dichotomized classification of high-degree (scale, 3 and 4) and low-degree (scale, 0–2) PVS^20^.

### PET acquisition and analysis

PiB was manufactured and handled at the PET center, NTUH, Taipei, Taiwan (specificity activity: 39 ± 19 GBq/μmol). All PET scanning was performed within 3 months after acquisition of the MRI. Static PET/CT scans (discovery ST; GE Healthcare, Waukesha, WI) were acquired in three-dimensional mode for 30 min starting 40 min after the injection of 10 mCi ^11^C-PiB. PET data were reconstructed with ordered set expectation maximization (five iterations; 32 subsets; post filter, 2.57) and corrected for attenuation. Each PiB PET image was realigned, resliced, and manually co-registered to a standardized CT template using PMOD software, as previously described^8,15,19^. The PET data were semi-quantitatively analyzed and expressed as the average mean standardized uptake value ratios (SUVRs) of the regions of interest using the cerebellar cortex as a reference region. The regions of interest in these spatially normalized images included the frontal, temporal, parietal, and occipital lobes, as defined in the Automated Anatomical Labeling Atlas. Areas of macrobleeds were manually excluded from the SUVR analyses, and the parameters in the specific regions of interest were determined using the ICH-free hemisphere.

### Statistical analysis

Categorical variables are presented as percentages and continuous variables are presented as mean ± SD or median (interquartile range) based on their distribution. Baseline demographics, clinical, and neuroimaging variables were compared between patients with CAA and HA using the Mann–Whitney *U*-test for continuous variables and Fisher’s exact test for categorical variables. We built multivariable logistic regression models to search for independent associations between CAA/HA and the lobar CMB topography (strictly intracortical/juxtacortical CMBs or subcortical CMBs); model 1 was adjusted for age and sex and model 2 was adjusted for age, sex, and other SVD neuroimaging markers. Additionally, we used the Youden index to determine best cutoff values for the number of lobar CMBs in each category (intracortical, intracortical/juxtacortical, subcortical) to differentiate CAA and HA. The diagnostic value, including sensitivity, specificity, positive predictive value, negative predictive value and the area under curve (AUC), was determined.

To further confirm our hypothesis that CAA is more frequently associated with intracortical lobar CMB topography, we compared the global and regional PiB SUVRs between patients with and without intracortical CMBs and between patients with and without subcortical CMBs using the Mann–Whitney *U*-test. The correlations between amyloid retention and the numbers of intracortical or subcortical CMBs were investigated using Spearman’s correlation and partial correlation analysis for age adjustment. All statistical analyses were performed using SPSS version 19 (IBM Corp., Armonk, NY, USA). All tests of significance were two-tailed with the threshold for significance defined as *P* < 0.05.

### Ethical approval

This study was performed with the approval of the institutional review board (201903069RINB) of National Taiwan University Hospital (NTUH) and in accordance with their guidelines. Written informed consent was obtained from all patients or their families in this study.

## Results

This analysis included 30 patients with CAA and 61 patients with HA (Table 1). Compared to patients with HA, patients with CAA were older (74.7 ± 9.4 vs. 63.0 ± 11.6 years, *P* < 0.001), more frequently female (60.0% vs. 26.2%, *P* = 0.003), had a higher prevalence of primary lobar hematoma (96.7% vs. 45.9%, *P* < 0.001), and had a lower rate of chronic hypertension (63.3% vs. 93.4%, *P* = 0.003). There were no significant differences in the total CMB count (4.0 [3–12] vs. 13.0 [6–29], P = 0.379) or lobar CMB count (3.0 [2–14] vs. 7.0 [2–18], P = 0.663) between groups.

**Table 1:** Comparison of demographics and neuroimaging markers between patients with CAA and HA.

	Cerebral amyloid angiopathy (*n* = 30)	Hypertensive arteriolosclerosis (*n* = 61)	*P* value
Female, %	18 (60.0%)	16 (26.2%)	0.003
Age, years	74.7 ± 9.4	63.0 ± 11.6	< 0.001
Lobar ICH, %	29 (96.7%)	28 (45.9%)	< 0.001
Hypertension	19 (63.3%)	57 (93.4%)	0.003
Diabetes	5 (16.7%)	12 (19.7%)	0.784
Hyperlipidemia	6 (20.0%)	20 (32.8%)	0.229
Lobar CMB subtype			
Intracortical lobar CMBs	24 (80.0%)	31 (50.8%)	0.011
Juxtacortical lobar CMBs	22 (73.3%)	51 (83.6%)	0.272
Subcortical lobar CMBs	4 (13.3%)	37 (60.7%)	< 0.001
Number of CMBs			
Total CMBs	4.0 (3–12)	13.0 (6–29)	0.379
Lobar CMBs	3.0 (2–14)	7.0 (2–18)	0.663
Intracortical lobar CMBs	2.0 (1–5)	1.0 (0–2)	< 0.001
Juxtacortical lobar CMBs	2.0 (0–5)	3.0 (1–6)	< 0.001
Subcortical lobar CMBs	0.0 (0–0)	1.0 (0–2)	0.003
Deep CMBs	0.0 (0–0)	4.0 (1–7)	< 0.001
WMH volume	11.4 (7.1–19.4)	17.4 (7.3–30.7)	0.017
Presence of Lacunes, %	13 (43.3%)	44 (72.1%)	0.011
Cortical superficial siderosis (cSS), %	12 (40.0%)	5 (8.2%)	0.001
MRI-visible perivascular spaces			
Centrum semiovale > 20	21 (70.0%)	25 (41.0%)	0.014
Basal ganglia > 20	10 (33.3%)	33 (54.1%)	0.076
Global PiB SUVR (IQR)	1.5 (1.3–1.6)	1.1 (1.0–1.2)	< 0.001

Values are mean (± standard deviation), median (interquartile range), or number (percentage).

*CMBs* cerebral microbleeds, *IQR* interquartile range, *PiB* 11C-Pittsburgh Compound B, *SUVR* standardized uptake value ratio, *WMH* white matter hyperintensity.

We next investigated the prevalence of each subtype of lobar CMB between patients with CAA and HA. The inter-rater agreements for detecting the presence of intracortical CMBs and subcortical CMBs were good (*k*, 0.85 [0.56–1] and 0.74 [0.41–1], respectively). Patients with CAA had a higher prevalence of intracortical CMBs (80.0% vs. 50.8%, *P* = 0.011) and lower prevalence of subcortical CMBs (13.3% vs. 60.1%, *P* < 0.001) compared to patients with HA. In addition, 75% of the included CAA patients with intracortical CMBs had both intracortical and juxtacortical CMBs. Therefore, a strictly intracortical CMB distribution (i.e., the absence of juxtacortical or subcortical CMBs) was not a sensitive imaging marker for CAA in our cohort. We chose to combine intracortical and juxtacortical CMBs and define “strictly intracortical/juxtacortical CMBs” to classify patients with an absence of subcortical CMBs. Strictly intracortical/juxtacortical lobar CMBs were more frequently found in patients with CAA compared to patients with HA (86.7% vs. 39.3%, *P* < 0.001).

The other SVD neuroimaging markers are compared in Table 1. CAA was significantly associated with cortical superficial siderosis (40.0% vs. 8.2%, *P* = 0.001), high-degree CSO-PVS (70.0% vs. 41.0%, *P* = 0.014), lower WMH volume (11.4 [7.1–19.4] vs. 17.4 [7.3–30.7], *P* = 0.017), a lower prevalence of lacunes (43.3% vs. 72.1%, *P* = 0.011), and a higher global PiB SUVR (1.5 [1.3–1.6] vs. 1.1 [1.0–1.2], *P* < 0.001).

We built logistic regression models to confirm the independent associations between the subtypes of lobar CMB and subtype of microangiopathy (Tables 2, 3). Strictly intracortical/juxtacortical CMBs were significantly associated with CAA after adjustment for age and sex (OR 10.8, 95% CI 2.8–41.4, *P* = 0.001, Table 2) and in the full model further adjusted for CAA-related neuroimaging markers including cortical superficial siderosis, CSO-PVS, and WMH volume (OR 18.9, 95% CI 1.9–191.4, *P* = 0.013, Table 2). On the other hand, the presence of subcortical CMBs was associated with HA (odds ratio [OR] 10.8, 95% CI 2.8‒41.4, *P* = 0.001, Table 3) after adjustment for age and sex, and this association remained significant in the full model adjusted for age, sex and hypertensive neuroimaging markers of BG-PVS, lacune, and WMH volume (OR 10.9, 95% CI 1.8–68.1, *P* = 0.010, Table 3).

**Table 2:** Multivariable models of the ability of neuroimaging markers to predict CAA ICH.

CAA ICH	Model 1 (age, sex)		Model 2 (full model)	
	Odds ratio (95% confidence interval)	*P* value	Odds ratio (95% confidence interval)	*P* value
Strictly intracortical/juxtacortical CMBs	10.8 (2.8–41.4)	0.001	18.9 (1.9–191.4)	0.013
Cortical superficial siderosis	–	–	2.5 (0.3–20.4)	0.398
CSO-PVS > 20	–	–	10.7 (1.7–69.7)	0.013
WMH volume, per 10 mL	–	–	0.5 (0.3–1.1)	0.101

*CMB* cerebral microbleed, *CSO* centrum semiovale, *PVS* perivascular spaces, *WMH* white matter hyperintensity.

**Table 3:** Multivariable models of the ability of neuroimaging markers to predict non-CAA ICH.

Non-CAA ICH	Model 1 (age, sex)		Model 2 (full model)	
	Odds ratio (95% confidence interval)	*P* value	Odds ratio (95% confidence interval)	*P* value
Subcortical CMBs	10.8 (2.8–41.4)	0.001	10.9 (1.8–68.1)	0.010
BG-PVS > 20	–	–	1.5 (0.4–6.3)	0.572
Lacunes	–	–	2.5 (0.6–9.6)	0.190
WMH volume, per 10 mL	–	–	1.8 (0.9–3.6)	0.119

*CMB* cerebral microbleed, *BG* basal ganglia, *PVS* perivascular spaces, *WMH* white matter hyperintensity.

The diagnostic value of the number of lobar CMBs in each category (intracortical, intracortical/juxtacortical, subcortical) for differentiation of CAA and HA is presented in the Supplementary Table. The best diagnostic accuracy was achieved for the number of subcortical lobar CMBs using a cutoff value of < 1 (AUC 0.76, 95% CI 0.664–0.856), suggesting that an absence of subcortical lobar CMBs (i.e., a strictly intracortical/juxtacortical CMB pattern) provided a sensitivity of 86.7% (95% CI 69.3–96.2) and a specificity of 60.7% (47.3–72.9) in our study cohort.

We next compared amyloid deposition between patients with the different subtypes of lobar CMBs (Table 4). Patients with intracortical CMBs had higher global and frontal amyloid retention compared to patients without intracortical CMBs (global SUVR: 1.16 [1.07–1.50] vs. 1.05 [1.01–1.14], *P* = 0.013; frontal SUVR: 1.18 [1.04–1.48] vs. 1.01 [0.96–1.17], *P* = 0.006). On the contrary, patients with subcortical CMBs had lower global and frontal amyloid retention compared to patients without subcortical CMBs (i.e., strictly intracortical/juxtacortical CMBs) (global SUVR: 1.08 [1.02–1.19] vs. 1.15 [1.05–1.52], *P* = 0.039; frontal SUVR: 1.05 [0.96–1.19] vs. 1.17 [1.00–1.53], *P* = 0.048). In correlation analysis, the global PiB SUVR was positively correlated with the number of intracortical CMBs (Spearman’s rho = 0.39, *P* < 0.001) and negatively correlated with the number of subcortical CMBs (Spearman’s rho = − 0.25, *P* = 0.018). The associations between global amyloid retention and the numbers of intracortical CMBs and subcortical CMBs remained significant in the partial correlation analysis adjusted for age (r = 0.31, *P* = 0.003 for intracortical CMBs; r = − 0.25, *P* = 0.019 for subcortical CMBs).

**Table 4:** Comparison of amyloid retention between patients with and without intracortical CMBs or subcortical CMBs.

	Intracortical CMB (+) (*n* = 55)	Intracortical CMB (−) (*n* = 36)	*P* value	Subcortical CMB (+) (*n* = 41)	Subcortical CMB (−) (*n* = 50)	*P* value
Global PiB SUVR	1.16 (1.07–1.50)	1.05 (1.01–1.14)	0.013	1.08 (1.02–1.19)	1.15 (1.05–1.52)	0.039
Frontal PiB SUVR	1.18 (1.04–1.48)	1.01 (0.96–1.15)	0.006	1.05 (0.96–1.19)	1.17 (1.00–1.53)	0.048
Occipital PiB SUVR	1.21 (1.12–1.45)	1.13 (1.08–1.24)	0.096	1.15 (1.10–1.23)	1.22 (1.10–1.47)	0.082

Values are median (interquartile range). *CMB* cerebral microbleed, *PiB* 11C-Pittsburgh Compound B, *SUVR* standardized uptake value ratio.

## Discussion

This study tested the hypothesis that cerebrovascular amyloid pathology is associated with intracortical lobar CMBs whereas deep perforator arteriolosclerosis is more strongly related to subcortical lobar CMBs. Our analysis indicates that CAA is associated with strictly intracortical/juxtacortical lobar CMBs while HA is associated with subcortical lobar CMBs. These findings imply that CAA leads to lobar CMBs that are mainly restricted to intracortical or juxtacortical regions, and less frequently extend to subcortical white matter. In contrast, the presence of subcortical CMBs probably results from underlying deep perforator arteriolosclerosis. These findings were confirmed by amyloid PET imaging, which showed that amyloid burden positively correlated with intracortical CMBs, but exhibited an inverse correlation with subcortical lobar CMBs. Taken together, our results indicate that patients with strictly intracortical/juxtacortical CMBs are more likely to harbor an underlying CAA pathology, whereas the presence of subcortical CMBs may imply underlying arteriolosclerosis due to hypertension and other vascular risk factors.

The diagnosis of the underlying subtype of SVD in spontaneous ICH depends heavily on conventional MRI markers, especially the location of ICH and CMB topography (lobar vs. deep)^22^. Lobar CMBs can occur in the absence of CAA^10^, suggesting that more effort is needed to precisely characterize the differences between lobar CMBs attributed to CAA or arteriolosclerosis. In this study, we developed a neuroimaging approach to categorize lobar CMBs according to their location relative to the cortex using SWI and the corresponding high-resolution T1-weighted MRI; these sequences are feasible in most clinical settings. The inclusion of non-hemorrhagic MRI features in the recently published Boston criteria v2.0 improved the diagnostic sensitivity for diagnosis of CAA, but at the expense of a slight drop in specificity^5^. Our study indicates that consideration of the anatomical location of lobar CMBs may increase the specificity and reduce the false positive rate of the current clinical criteria. Additionally, the lobar CMB categories that reflect different subtypes of SVD may have the potential to predict patient outcomes in the SVD population, and this approach warrants validation in future prospective studies of different cohorts.

One important finding of this study is that the presence of subcortical CMBs—the subtype of lobar CMB completely located in the subcortical white matter without reaching the cortex—is independently associated with HA. This association implies subcortical CMBs are probably not the result of underlying CAA. These findings are supported by a recent neuropathological study based on ex vivo 7-T MRI, which showed that lobar CMBs were predominantly located in the superficial layers of the cortex in CAA-related lobar ICH, but in the deeper layers of the cortex in non-lobar ICH^23^. Although the authors did not detect a difference in the rate of subcortical lobar CMBs between patients with lobar and non-lobar ICHs, their results imply that CAA and arteriolosclerosis tend to affect different vessels in the cortex. In line with this finding, an earlier report showed that 77% of the lobar CMBs in patients with CAA were located in the more superficial layers of the cortex^24^. A recently published histopathological study found CMBs discovered in false-positive CAA were mostly located along the culprit vessels with moderate to severe arteriolosclerosis, and these CMBs were located in juxtacortical or subcortical white matter rather than cortical regions^25^. Together, the existing evidence suggests that CAA tends to affect very superficial cortical vessels, while HA may more frequently involve the terminal perforators. This evidence also implies the precise location of CMBs could potentially act as an additional diagnostic marker and enhance the current diagnostic accuracy for CAA.

In terms of clinical significance, our study highlights the potential of assessing the subtypes of lobar CMB to enhance the diagnosis of CAA—especially in difficult case scenarios where CAA is a consideration but the diagnosis is not covered by current diagnostic criteria^5^. The two predominant small vessel pathologies, CAA and HA, have been shown to frequently coexist with each other^8,26,27^. Mixed lobar and deep CMBs are commonly encountered in patients with spontaneous ICH; however, neuroimaging markers to identify coexisting CAA are still very limited^7,8^. Therefore, supportive pathological data or in vivo molecular imaging are needed to better characterize the contribution of CAA to lobar CMBs. One strength of the current study is the use of amyloid PET as a surrogate marker for cerebrovascular amyloid burden^12,28^, which enabled confirmation of the coexistence and severity of CAA for cases with mixed lobar and deep bleeds, as shown in our previous report^8^. Using in vivo PiB PET, we observed higher amyloid retention in patients with intracortical CMBs than patients without intracortical CMBs. In addition, the cortical amyloid load positively correlated with the number of intracortical CMBs and negatively correlated with the number of subcortical CMBs. These results re-enforce our hypothesis that CAA is associated with intracortical CMBs but not subcortical CMBs, at least from the perspective of molecular imaging.

This study has several limitations. First, parenchymal as well as vascular amyloid deposition can also be a source of high PiB binding, and we could not completely exclude the confounding effects of Alzheimer’s pathology in the current study. Another major limitation is the low image resolution of lobar CMBs on 3 T MRI and the blooming effect due to susceptibility artifacts^13^. We overcame this limitation by categorizing the lobar CMBs using the corresponding thin-slice T1-weighted images, in which the exact location of the lobar CMB could be better defined. However, our approach still prevents further localization of intracortical CMBs in the superficial or deep layers of the cortex. Moreover, a proportion of lobar CMBs were also categorized as the juxtacortical subtype, and the significance of this subtype remains to be undetermined. This issue could be a result of the limited resolution of 3 T MRI in our study. Future studies that employ ultra-high field MRI combined with in vivo amyloid imaging are needed to address this issue. On the other hand, 1.5 T MRI is more widely used in clinical settings, and differentiation of cortical and subcortical regions may be even more difficult using this imaging modality. The utility of 1.5 T MRI for determination of the anatomical location of lobar CMBs needs to be confirmed in further studies. Third, our study was performed with the v1.5 Boston criteria rather than the newest v2.0. However, all of our included patients had symptomatic ICH with at least one lobar CMB, and thus met the criteria of probable CAA in both v1.5 and v2.0 of the Boston criteria. Therefore, patient selection and our study findings would not be affected by this issue. Similarly, the SVD MRI markers were assessed based on STRIVE instead of the updated STRIVE-2. However, as the definitions of essential SVD markers have not changed, the updated criteria would not affect the neuroimaging assessment in our study. Lastly, due to the lack of more specific biomarkers for arteriolosclerosis in cortical vessels, we cannot precisely evaluate the interaction between CAA and HA; however, these pathologies appear to share some common pathophysiological pathways^23,29^. The interaction of these pathologies represents an interesting target for future research that may provide important mechanistic insights into the underlying brain lesions caused by mixed CAA and arteriolosclerosis.

## Conclusion

We provide neuroimaging evidence to indicate that CAA is more closely associated with strictly intracortical/juxtacortical lobar CMBs than subcortical lobar CMBs. Patients with strictly intracortical/juxtacortical lobar CMBs have underlying CAA whereas patients with subcortical lobar CMBs are likely to harbor underlying arteriolosclerosis. Thus, categorization of lobar CMBs based on their anatomical location could help to more precisely delineate the underlying forms of SVD and may therefore have important clinical implications.

### Supplementary Information

Supplementary Tables.

## Data Availability

All data from this article are being held within NTUH and will be shared with qualified investigators on request. Please contact Dr. Hsin-Hsi Tsai (hsinhsi@ntu.edu.tw) to request the data.

## Abbreviations

AUC: Area under curve
BG: Basal ganglion
CAA: Cerebral amyloid angiopathy
CMB: Cerebral microbleed
CSO: Centrum semiovale
HA: Hypertensive arteriolosclerosis
ICH: Intracerebral hemorrhage
PiB: ^11^C-Pittsburgh compound B
PVS: Perivascular space
SUVR: Standardized uptake value ratio
SVD: Small vessel disease
SWI: Susceptibility-weighted imaging
WMH: White matter hyperintensity
