# Supplementary material for: Differences in lobar microbleed topography in cerebral amyloid angiopathy and hypertensive arteriopathy
Source: Sci Rep. 2024 Feb 15;14:3774. doi: 10.1038/s41598-024-54243-1 (PMC10866968; doi:10.1038/s41598-024-54243-1)
Supplement: Supplementary file 1 — Supplementary Tables. [file 41598_2024_54243_MOESM1_ESM.docx]

**Supplementary Table. Diagnostic performance of intracortical, strictly intracortical/juxtacortical and subcortical CMB number in predicting CAA-ICH.**

| \|  \| **Intracortical CMB number** \| **Intracortical/juxtacortical CMB number** \| **Subcortical CMB number** \| \| --- \| --- \| --- \| --- \| \| **Cutoff value** \| ≥1 \| ≥2 \| <1 \| \| **Sensitivity, %** \| 80.0 (61.4-92.3) \| 76.7 (57.7-90.1) \| 86.7 (69.3-96.2) \| \| **Specificity, %** \| 49.2 (36.1-62.3) \| 27.9 (17.2-40.8) \| 60.7 (47.3-72.9) \| \| **AUC** \| 0.677 (0.560-0.794) \| 0.525 (0.395-0.656) \| 0.760 (0.664-0.856) \| \| **PPV, %** \| 43.6 (36.3-51.2) \| 34.3 (28.9-40.2) \| 52.0 (43.5-60.4) \| \| **NPV, %** \| 83.3 (70.1-91.5) \| 70.8 (53.1-83.9) \| 90.2 (78.4-95.9) \|   AUC: Area under curve; CMB: Cerebral microbleed; CAA: Cerebral amyloid angiopathy; NPV: negative predictive value; PPV: positive predictive value; |
| --- | --- | --- | --- | --- | --- | --- | --- | --- | --- | --- | --- | --- | --- | --- | --- | --- | --- | --- | --- | --- | --- | --- | --- | --- | --- | --- | --- | --- |
